# Hepatitis B surface gene 145 mutant as a minor population in hepatitis B virus carriers

**DOI:** 10.1186/1756-0500-5-22

**Published:** 2012-01-10

**Authors:** Haruki Komatsu, Ayano Inui, Tsuyoshi Sogo, Yasuhiro Konishi, Akihiko Tateno, Tomoo Fujisawa

**Affiliations:** 1 Division of Hepatology and Gastroenterology, Department of Pediatrics, Eastern Yokohama Hospital, 3-6-1 Simosueyoshi Tsurumi, Yokohama, Kanagawa, Japan; 2 Department of Obstetrics & Gynecology, Eastern Yokohama Hospital, Yokohama, Kanagawa, Japan; 3 Department of Pediatrics, Toho University Sakura Medical Center, Sakura, Chiba, Japan

## Abstract

**Background:**

Hepatitis B virus (HBV) can have mutations that include the *a *determinant, which causes breakthrough infection. In particular, a single mutation at amino acid 145 of the surface protein (G145) is frequently reported in the failure of prophylactic treatment. The aim of this study was to evaluate the frequency of the *a *determinant mutants, especially the G145 variant, in Japan, where universal vaccination has not been adopted.

**Methods:**

The present study was a retrospective study. The study cohorts were defined as follows: group 1, children with failure to prevent mother-to-child transmission despite immunoprophylaxis (n = 18, male/female = 8/10, age 1-14 years; median 6 years); group 2, HBV carriers who had not received vaccination or hepatitis B immunoglobulin (n = 107, male/female = 107, age 1-52 years; median 16 years). To detect the G145R and G145A mutants in patients, we designed 3 probes for real-time PCR. We also performed direct sequencing and cloning of PCR products.

**Results:**

By mutant-specific real-time PCR, one subject (5.6%) was positive for the G145R mutant in group 1, while the G145 mutant was undetectable in group 2. The *a *determinant mutants were detected in one (5.6%) of the group 1 subjects and 10 (9.3%) of the group 2 subjects using direct sequencing, but direct sequencing did not reveal the G145 mutant as a predominant strain in the two groups. However, the subject who was positive according to the mutant-specific real-time PCR in group 1 had overlapped peaks at nt 587 in the electropherogram. In group 2, 11 patients had overlapped peaks at nt 587 in the electropherogram. Cloning of PCR products allowed detection of the G145R mutant as a minor strain in 7 (group 1: 1 subject, group 2: 6 subjects) of 12 subjects who had overlapped peaks at nt 587 in the electropherogram.

**Conclusions:**

The frequency of the *a *determinant mutants was not high in Japan. However, the G145R mutant was often present as a minor population in children and adults. HBV carriers might have the *a *determinant mutants as a minor form.

## Background

Hepatitis B virus (HBV) variants with mutations in the *a *determinant frequently emerge under immunological pressure induced by the HB vaccine or HB immunoglobulin (HBIG)[1-4]. Although the mechanism of the emergence of *a *determinant mutants remains unclear, preexisting mutants as a minor population or as the predominant population could survive, replicate, and cause a breakthrough infection after the host receives the HB vaccine or HBIG. Japan continues to implement an HBV immunization strategy that targets high-risk groups rather than instituting a universal vaccination program. There is no immune pressure induced by universal vaccination, and thus knowing the prevalence of mutants in Japan will be useful for clarifying the mechanism of the emergence of mutants.

Of various mutants with the *a *determinant, the mutant with a single mutation at the 145th amino acid of the hepatitis B surface antigen (HBsAg) has been frequently reported to cause the failure of prophylaxis in mother-to-child transmission [5-10]. Thus, it is indispensable to clarify the frequency of the *a *determinant mutants when developing the future vaccine strategy in Japan. The aim of this study was to evaluate the frequency of the *a *determinant mutants in HBV carriers. In particular, we focused on the G145 variant as a minor strain. Mutant-specific real-time polymerase chain reaction (PCR) and direct sequencing were performed. Moreover, cloning of PCR products was used to investigate the presence of the G145variant as a minor strain.

## Methods

### Patients

The present study was a retrospective study. The study cohorts consisted of the following two groups: group 1, children with failure to prevent mother-to-child transmission despite prophylaxis; group 2, HBV carriers who had not received the HB vaccination or HBIG. A total of 18 children (male/female = 8/10, age: 1-14 years, median 6, genotype A/B/C = 0/0/18) were referred to our institute to failure of prophylactic treatment with the HB vaccine or HBIG in mother-to-child transmission. They belonged to the failure of prophylactic treatment group (group 1). A total of 107 chronically infected patients (male/female = 47/60, age: 1-9 years; n = 19, age 10-19 years; n = 54, age: 20-52 years; n = 34, genotype A/B/C = 1/14/92) were followed in our institute. They had no history of receiving the HB vaccine or HBIG. These subjects belonged to group 2. The patients' characteristics by group are shown in Table 1. Informed written consent for study participation was obtained from all patients or their parents. The study protocol was approved by the ethics committees of Yokohama Eastern Hospital.

**Table 1:** Patient characteristics by group

	Failure of prophylactic treatment for mother-to-child transmission	Chronic HBV infection without HBIG or HB vaccine
	**n = 18**	**n = 107**

Gender, male/female	8/10	47/60

Age	1-14 yr. (median, 6)	1-52 yr. (median, 16)

Genotype A/B/C	0/0/18	1/14/92

HBeAg (mother)	16 (15, 3 unknown)	60

HBV DNA levels in blood, log copies/mL	5.6 - > 8.8 (median, 8.6)	2.1 - > 8.8 (median, 4.9)

### HBV DNA extraction and quantification of HBV DNA in serum

HBV DNA was extracted from 200 μL of serum using QIAamp DNA Blood Mini kit (QIAGEN, Hilden, Germany). The real-time PCR was performed for quantification using the genotype-independent real-time PCR method described previously [11]. The PCR assay was performed in a MX3000P (Stratagene), and the results were analyzed with MxPro software (version 3.0). The lower detection limit was > 100 copies/mL. All assays were carried out in duplicate with negative control samples.

### Real-time PCR for the G145R and G145A mutants

To detect the G145R and G145A mutants as minor strains in patients, we designed 3 probes based on the sequences of the G145R and G145A mutants [12]. The primers used were 5'-GAT TCC TGC TCA AGG AAC CTC-3' (forward; nt 529-549) and 5'-CGA AAG CCC AGG ATG ATG-3' (reverse; nt 612-629). The point mutation at nt 587 (G145R, mutant 1: G to A, mutant 2: G to C) and nt 588 (G145A, mutant 3: G to C) were applied to the sequence of the probes. The following probes (nt 571-591) were used for mutant-specific real-time PCR: wild type: 5'-FAM-TACAAAACCTTCGGACGGAAACTGC-TAMRA-3'; mutant 1: 5'-FAM-TACAAAACCTTCGGACAGAAACTGC-TAMRA-3'; mutant 2: 5'-FAM-TACAAAACCTTCGGACCGAAACTGC-TAMRA-3'; mutant 3: 5'-FAM-TACAAAACCTTCGGACGCAAACTGC-TAMRA-3'. Nucleotide positions were designated on the basis of nucleotide sequences from genotype C (GenBank/EMBL accession number AB300361). PCR was performed in a 50-μL reaction mixture containing 25 μL TaqMan Universal PCR master mix (Applied Biosystems) with 0.2 μM primers, 0.1 μM probes, and 10 μL extracted DNA. The PCR program consisted of an initial pre-cycle incubation at 50°C for 2 min and 95°C for 10 min, followed by 50 cycles of 95°C for 15 s and 60°C for 1 min. All assays were carried out in duplicate with negative control samples.

### Sequencing and cloning

All HBV DNA samples were amplified by nested PCR using 2 primer pairs with sequences corresponding to the surface region of the HBV genome, which encompassed *a *determinant region. We used the following primers: outer sense: ACAGAGTCTAGACTCGTGGT (nt 241-260); outer antisense: AAAGCCCTACGAACCACTGA (nt 694-713); inner sense: GGACTTCTCTCAATTTTCTAGGG (nt 261-283); inner antisense: CAAATGGCACTAGTAAACTGAGC (nt 670-692). Amplification was performed by nested PCR in a 50-μl reaction mixture containing 25 pmol of each primer and 2.5 U of Taq DNA polymerase (TaKaRa Ex Taq, Takara Bio, Shiga, Japan). The first round of amplification was performed for 35 cycles (denaturation at 94°C for 30 s, annealing at 55°C for 30 s, and extension at 72°C for 1 min) with the external primers. In the second round of PCR, 1 μM of the first-round PCR products was submitted to a second round of PCR using the internal primers. The second-round PCR program was the same as that for the first round. PCR products were cloned and sequenced using a TOPO TA cloning kit for sequencing (Invitrogen, Carlsbad, CA). DNA sequences were determined using the Applied Biosystems 3730xl DNA Analyzer (Applied Biosystems, Foster City, CA).

## Results

### Sensitivity of mutant-specific real-time PCR

**Figure 1:**
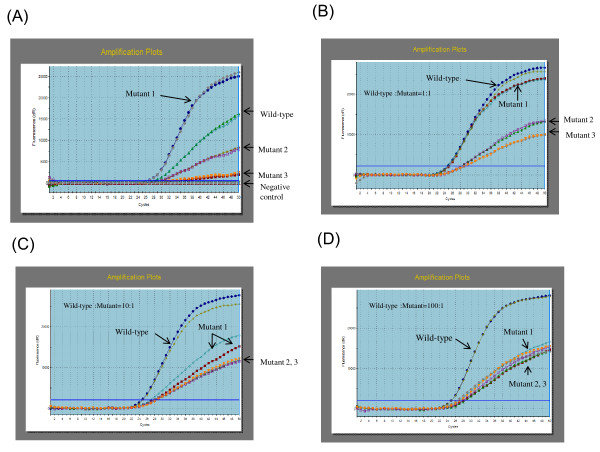
**Mutant-specific real-time PCR was performed in duplicate for each probe**. (**A**) HBV DNA from serum with an escape mutant (single mutation at nt 587, A to G) was amplified using the wild-type and mutant 1, 2, and 3 probes. (**B**) Constructed plasmid mutant-type (single mutation at nt 587 G to A) DNA and wild-type DNA were mixed. The amount of the mutant-type plasmid DNA was equal to that of the wild-type DNA. The curves of the amplification plots are indicated by arrows. (**C**) Constructed plasmid mutant-type (single mutation at nt 587 G to A) DNA and wild-type DNA were mixed. The mutant:wild ratio in plasmid DNA was 1:10. (**D**) Constructed plasmid mutant-type (single mutation at nt 587 G to A) DNA and wild-type DNA were mixed. The mutant:wild ratio in plasmid DNA was 1:100.

We evaluated the sensitivity and specificity of mutant-specific real-time PCR (Figure 1). HBV DNA from clinical serum samples in which we had confirmed a mutation with G145R (at nt 587 G to A, genotype C, the levels of HBV DNA: 5.7 log copies/mL) was amplified using the mutant-specific real-time PCR in duplicate. The amplification signals were detected for HBV DNA from serum with mutant G145R in the wild probe and the mutant probe. However, cycle threshold (Ct) values in the mutant probe (mutant 1, Ct values = 26.42 and 26.57) were lower than those in the wild probe (Ct values = 27.76 and 27.99), as shown Figure 1A. In addition, the amplification curves in the mutant 1 probe showed a steeper upward slope. Although the mutant primer detected a weak signal of wild-type HBV DNA, these findings suggested that the mutant primer could clearly identify the mutant with a point mutation at nt 587 G to A if the mutant was the predominant strain.

Next, we assessed whether the specific probe could distinguish the G145R mutant as a minor strain from the predominant wild-type strain (Figure 1B-C). Constructed plasmid mutant-type (a point mutation at nt 587 G to A) DNA and wild-type DNA were mixed. The results of real-time PCR using 4 probes for mixed DNA at mutant:wild ratios of 1:1, 1:10, and 1:100 are shown in Figures 1B-C, and 1D, respectively. At the 1:1 ratio, the amplification curve of the mutant 1 probe showed the same Ct values and similar steep slopes (wild-type: Ct values = 24.53 and 24.71; mutant 1: Ct values = 24.86 and 24.27) (Figure 1B). At the 1:10 ratio, however, the amplification curve of the mutant probe showed a less steep upward slope compared to that of the wild-type probe, and the Ct values were greater (wild-type: Ct values = 24.52 and 24.76; mutant 1: Ct values = 26.60 and 27.27; mutant 2: Ct values = 27.61 and 27.89; mutant 3: Ct values = 27.58 and 28.36) (Figure 1C). At the 1:100 ratio, there was no difference in the Ct values or in the shape of the amplification curves among these mutant-specific probes, and it was impossible to identify the mutant with a point mutation at nt 587 G to A (wild-type: Ct values = 24.74 and 24.91; mutant 1: Ct values = 27.65 and 27.75; mutant 2: Ct values = 28.04 and 28.12; mutant 3: Ct values = 26.95 and 27.17) (Figure 1D). These findings suggested that the mutant probe could detect the G145R mutant representing as little as 10% of the wild-type population. Therefore, if the Ct value in a mutant-type probe was the same as or lower than that in the wild-type probe, the sample was considered to be positive for the mutant-specific real-time PCR.

### Positive rate of mutant-specific real-time PCR

#### Chronically infected children despite receiving immunoprophylaxis

**Figure 2:**
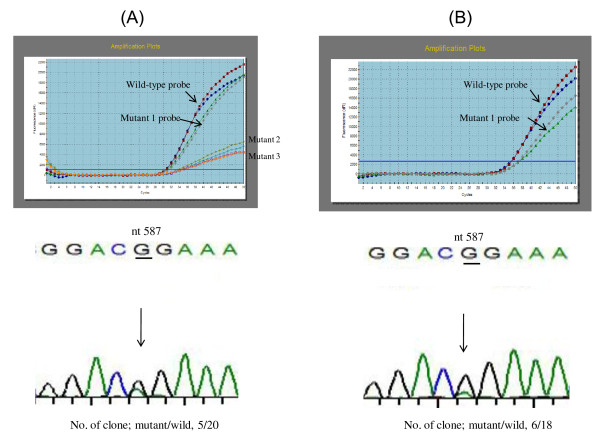
**Serum HBV DNA from a girl who became positive for HBsAg and negative for anti-HBs despite immunoprophylaxis was amplified by mutant-specific real-time PCR in duplicate**. (**A**) Before antiviral therapy (May 2007), the Ct values of the amplification plots in the mutant 1 probe were the same as those for the wild-type probe. In addition, the amplification plots of both probes were similar in the upward slopes. Although the signals of the mutant 2 and 3 probes were detectable, the curves of the amplification plots were not steep. There was an overlapped peak at nt 587 in the electropherogram. The larger peak was G, and the smaller peak was A. Of the 20 clones, 5 had a mutation at nt 587 from G to A. (**B**) At the cessation of antiviral therapy (May 2009), the girl was negative for serum HBV DNA by real-time PCR. 4 months after the completion of antiviral therapy, however, she became positive again for serum HBV DNA. The signals of the mutant 1 probe as well as the wild-type probe were detected in the amplification plots (the mutant 2 and 3 probes were not used). The curves of the amplification plots were steep. There was an overlapped peak at nt 587 in the electropherogram. Of the 18 clones, six had a mutation at nt 587 from G to A.

The emergence of mutants is a well-known cause of the failure of immunoprophylactic treatment for mother-to-child transmission of HBV. Of the 18 children chronically infected despite receiving immunoprophylaxis, one (5.6%), a 10-year-old girl, was positive for the mutant-specific real-time PCR using the mutant 1 probe (Table 2). This child was also positive for the wild-type probe (Figure 2A).

**Table 2:** Frequency of nt 587 G to A (aa 145 Gly to Arg) mutants

	**no./total no**.	
	**Failure of prophylactic treatment for mother-to-child transmission**	**Chronic HBV infection without HBIG or HB vaccine**

Mutant specific real time PCR	1/18 (5.6)	0/107

Direct sequencing (major clone)	0/18	0/107

#### HBV carriers without HBIG or the HB vaccine

We examined 107 HBV carriers who had never received HBIG or the HB vaccine and found that none of them was positive for the mutant-specific real-time PCR (Table 2). The primers of the mutant-specific real-time PCR did not work in a patient infected with genotype A. Genotype from D to H were not available in this study.

### Direct sequencing

To confirm the results of the mutant-specific real-time PCR, we performed direct sequencing. Although the girl with failure of immunoprophylaxis was positive for the mutant 1 probe, the G145R mutant was not detected as a predominant strain in any patients (Table 2). In addition to the G145R mutant, the *a *determinant (aa 124-147) was evaluated using the direct sequencing. As a predominant strain, I/T126S mutant was detected in one (5.6%) of the 18 children with failure of prophylaxis. In addition, I/T126S (n = 4), I/T126V (n = 1), Q129L (n = 1), T131P (n = 2), M133T + T140I (n = 1), and S136Y (n = 1) mutants were detected as a predominant strain in 10 (9.3%) of the 107 HBV carriers who had not received the HB vaccine or HBIG.

### Detection of G145R mutant by cloning of PCR products

The mutant-specific real-time PCR (mutant 1 probe) could detect the G145R mutant in a child with failure of immunoprophylaxis. The amplification curves of this girl indicated that the G145R mutant strain and wild-type strain were coexisting (Figure 2A, upper, the serum sample was taken in May 2007). Consistent with the results of the mutant-specific real-time PCR, her electropherogram indicated that the wild-type HBV was the predominant strain and the G145R mutant was a minor strain. Of the 20 clones, 5 (25.0%) were G145R mutants (Figure 2A, lower) by PCR cloning technique. This finding suggested that the mutant-specific real-time PCR had the ability to detect 25% of minor strains in HBV populations. The girl was positive for HBeAg. Her serum ALT values had been elevated to twice the normal values for 6 years, and the levels of HBV DNA in her blood were more than 7 log copies/ml. She had received antiviral therapy (entecavir, 0.5 mg/day) for 48 weeks (between July 2007 and June 2008) and had become negative for serum HBV DNA by the completion of the therapy. 4 months after the completion of the therapy, however, serum HBV DNA became detectable. The mutant-specific real-time PCR and PCR cloning were performed again (Figure 2B, upper, the serum sample was taken in May 2009), and she was found to be positive for serum HBV DNA. PCR cloning revealed that 6 (33.3%) of 18 clones were G145R mutants (Figure 2B, lower).

The result of the PCR cloning for the G145R mutant in the girl with failure of immunoprophylaxis raised a question. In her sequence electropherogram, a larger peak and a smaller peak overlapped at nt 587 of HBsAg (Figure 2). The larger peak and the smaller peak were considered to represent the predominant strain and the minor strain, respectively. This finding suggested that the electropherogram could be useful for the detection of minor populations, so we investigated whether cloning of PCR products was more sensitive than mutant-specific real-time PCR for the detection of minor populations. Of all the patients in groups 1 and 2, 12 (group 1: F-16, group 2: CHB-2, -5, -26, -30, -42, -62, -67, -73, -94, -100, and -104) had overlapped peaks at nt 587 in the electropherogram. We thus performed cloning of PCR products in all of these patients except for F-16. Ten or more clones were sequenced. Of the 11 patients, 6 (CHB-26, -62, -67, -73, -94, and -104) had the G145R mutant as the minor strain (Table 3). All 6 of these subjects were HBV carriers with no history of HBIG or the HB vaccine. Of the 7 HBV carriers with the G145R mutant, including the girl with failure of immunoprophylaxis (F-16), 6 were positive for HBeAg (Table 3, upper). The remaining 5 patients (CHB-2, -5, -30, -42, and -100), who had overlapped peaks at nt 587 by sequencing, did not have the G145R mutant as a minor strain (Table 3, lower).

**Table 3:** Results of PCR cloning in patients who had overlapped peaks (G and A) at nt 587 in the electropherogram

**Patint ID**.	age (yr.)	HBsAg	HBeAg	HBV DNA in blood, log copies/mL	**no./total no**.	
					**Wild-type**	**Mutant-type (%)**

F-16	10	positive	positive	5.2	15/20	5/20 (25)

CHB-26	12	positive	positive	6.9	12/14	2/14 (14)

CHB-62	15	positive	negative	2.3	13/15	2/15 (13)

CHB-67	13	positive	positive	4.1	24/25	1/25 (4)

CHB-73	43	positive	positive	5.2	14/16	2/16 (13)

CHB-94	10	positive	positive	7.4	14/15	1/15 (7)

CHB-104	40	positive	positive	> 8.8	15/16	1/16 (6)

CHB-2	7	positive	negative	2.1	16/16	

CHB-5	14	positive	positive	4.9	30/30	

CHB-30	43	positive	positive	5.3	14/14	

CHB-42	24	positive	positive	8.7	28/28	

CHB-100	42	positive	positive	6.3	30/30	

## Discussion

In this study, the *a *determinant mutants were detected as predominant strains by direct sequencing in 9.3% of chronic hepatitis B patients who had not received the HB vaccine or HBIG.. This figure was comparable with that reported in a previous study (7.8%), which was conducted in Taiwan before the introduction of universal vaccination [13,14]. This finding indicates that the presence of the *a *determinant mutants was not common in Japanese children. However, previous studies showed a higher prevalence rate of the *a *determinant mutants in Japan. Ogura et al. reported that the *a *determinant mutants were detected in 24% (10/42) of unselected Japanese HBV carriers [15]. In addition, Takahashi et al. reported that 48% (19/40) of HBs-positive hepatocelluar carcinoma patients had the *a *determinant mutants in Japan [16]. Apart from Japan, Avellon et al. reported that the *a *determinant mutants were detected in 39% (106/272) of unselected carriers in Spain [17]. that HBV carriers. Presumably, the age (duration of infection), the degree of chronic hepatitis, and immunological selection by vaccine might have influenced the emergence of mutants. For instance, after the introduction of universal vaccination in Taiwan, the prevalence of the *a *determinant mutants was slightly increased and remained approximately 20% between 1989 and 2004 [13,14].

Direct sequencing showed that I/T126S [9,18,19], I/T126V [16], Q129L [20,21], T131P [22], M133T + T140I [13,14,21,23], and S136Y [24,25] mutants were present as a predominant strain in 107 HBV carriers. All of these mutants were reported in previous studies, but the pathogenicity of I/T126V, Q129L, T131P has not been confirmed. Among these mutants, the I/T126S mutant was the most frequent (4/107 = 4.7%) in the group 2 of the present study. Moreover, the I/T126S mutant was detected in a child with the prophylactic failure in group 1. In the previous study targeting unselected Spanish carriers, the prevalence rate of the I/T126S mutant was 0.4% (1/272)[17]. Compared with the previous study, the frequency of I/T126S of the present study is high. The I/T126S mutant has been frequently observed in the studies from Japan, in 12% (5/42) [15] and 13% (5/40)[16] of adult HBV carriers. In contrast, a mutation at the 127th amino acid of the hepatitis B surface antigen (genotype A:5.9%, genotype D: 8.8%) was the most frequently reported mutation in a Spanish study [17]. Genotypes A and E are predominant in Europe, whereas genotypes B and C prevail in Southeast Asia, including Japan. There is a possibility that the difference among HBV genotype could be associated with the frequency of the I/T126S mutant.

Before the present experiment was performed, we predicted that the mutant-specific real-time PCR technique would be able to more frequently detect the G145 mutant compared with sequencing and cloning. Using the constructed plasmid, the mutant-specific probe could detect 10% of mutants among wild-type virus in this study. Similarly, Zhang et al. reported that the real-time PCR method could detect 5% of mutants among wild-type virus [12]. The present study showed that the mutant-specific real-time PCR identified a child in whom the G145R mutant and wild-type virus were mixed. In this case, the number of the G145R mutant clones was 5 (25%) out of 20 clones. These findings proved that the mutant-specific probe could detect a minor mutant-type virus among viral populations in a clinical sample. However, we found that sequencing and cloning were more sensitive than the mutant-specific real-time PCR, although performance of these techniques required more time. Sequencing and cloning could detect much smaller populations of mutant-type virus, ranging from 6% to 14% of subpopulations in this study. If cloning of PCR products is carefully performed in every nucleotide of the *a *determinant, the prevalence of finding the *a *determinant mutant will be increased. Therefore, the *a *determinant mutant is present as a minor population in a high proportion of HBV carriers. This notion is consistent with the fact that selection immunological pressure such as the HB vaccine and HBIG allows the minor *a *determent mutant to become the predominant strain of the virus.

The virulence of the G145R mutant is indeterminate. The results of previous studies suggested that the G145R mutation reduced the ability of viral assembly and secretion [26,27]. In this study, however, the levels of serum transaminases had been elevated and the levels of HBV DNA had remained high for several years in the girl infected with the G145R mutant, which was detected by the mutant-specific real-time PCR. The levels of HBV DNA in the blood were not significantly lower in patients with the G145R mutant than in patients without the G145R mutant (data not shown). Although the G145R mutant was mixed with the predominant wild-type virus and viral replication was influenced by pre-core and basal core promoter mutation, these findings suggest that infection with the G145R mutant does not always promise a good prognosis. After treatment with entecavir, the G145R mutant as well as the wild-type virus appeared again in the girl.

Of the 18 the children with prophylactic failure in the present study, one had an *a *determinant mutant (I/T126S) as a predominant strain and one had an *a *determinant mutant (G145R) as a minor strain. This finding was consistent with previous studies [5,28,29]. The detection rate of *a *determinant mutation varied from 12% to 26% in children with prophylactic failure, indicating that the main cause of failure to prevent mother-to-child transmission was not the emergence of *a *determinant mutants. Recent studies suggest that high viral load in blood is closely related to the failure of prophylaxis with vaccine and HBIG [30-32].

## Conclusions

The *a *determinant mutants were not the predominant mutations in Japan. However, the G145R mutant was present as a minor population in children and adults. Numerous *a *determinant mutants could be lost in the mass of the wild-type virus.

## Abbreviations

HBV: Hepatitis B virus; HB vaccine: Hepatitis B vaccine; HBIG: Hepatitis B immunoglobulin; HBsAg: Hepatitis B surface antigen; HBeAg: Hepatitis B e antigen; PCR: Polymerase chain reaction; Anti-HBs: Antibodies against HBsAg

## Competing interests

The authors declare that they have no competing interests.

## Authors' contributions

HK contributed to the design of this study and drafted this manuscript. AI, TS, YK, AT, and TF participated in data collection and critical revision of the manuscript. All the authors concurred with the submission and will take responsibility for the manuscript.
